# Towards natural stand-up movement support: guiding higher-dimensional muscle activation using a Lower-DOF assistive chair

**DOI:** 10.3389/fbioe.2026.1771282

**Published:** 2026-03-06

**Authors:** Takahide Ito, Jun Morimoto, Qi An, Yuichi Nakamura, Jun-ichiro Furukawa

**Affiliations:** 1 Guardian Robot Project, RIKEN, Kyoto, Japan; 2 Graduate School of Informatics, Kyoto University, Kyoto, Japan; 3 Department of Human Engineered Environmental Studies, Graduate School of Frontier Sciences, University of Tokyo, Chiba, Japan; 4 Academic Center for Computing and Media Studies, Kyoto University, Kyoto, Japan; 5 Faculty of Systems Engineering, Wakayama University, Wakayama, Japan

**Keywords:** assistive chair, classification of assistive parameter, muscle activity inducement, muscle activity inference, sit-to-stand motion

## Abstract

Sit-to-stand (STS) assistance should not only reduce effort but also preserve or shape neuromuscular activity patterns. We propose a data-driven control strategy for an assistive chair with two degrees of freedom (vertical and horizontal seat motion) to infer desired multi-muscle activation during STS. The chair is parameterized by four binary variables (fast/slow vertical and horizontal velocities, and early/late onset timing for each axis), yielding 16 control combinations. Surface EMG from eight lower-limb muscles was collected from six healthy adult males across all control combinations (10 trials per condition). We extracted hundred-dimensional EMG features by segmenting STS into four phases and computing summary statistics per muscle and phase. Four $L_{1}$ -regularized logistic regression classifiers were trained to infer each control variable from EMG features, enabling a classifier-based statistical mapping from target EMG features to chair control parameters. The classifiers achieved F-scores of 0.96 and 0.99 for forward and upward speed, and 0.89 and 0.82 for forward and upward timing, respectively. In an offline evaluation, the estimated control parameters inferred EMG feature patterns significantly closer to the target than non-target parameter combinations. These results suggest that low-DoF seat motion can be used to modulate higher-dimensional muscle activation patterns during STS, providing a basis for future real-time and individualized assistive control.

## 1 Introduction

Assistive systems for humans have been developed for a wide range of applications to support mobility and n the context of an aging society. Among the various target motions, the sit-to-stand (STS) transition has received considerable attention (Frykberg and Häger, 2015). STS is performed frequently in daily life and requires substantial whole-body effort; adults reportedly perform STS more than 45 times per day (Bohannon, 2015). A decline in STS ability can therefore limit other functional movements and reduce quality of life (Slaughter et al., 2015), motivating the development of STS assist systems for supporting and compensating for frailty in older adults (Galli et al., 2008). However, while assistance can reduce the mechanical workload of STS, assistive devices have been shown to influence muscle activity profiles and motor behavior (Ito et al., 2023; Huo et al., 2022). Accordingly, STS assistance should ideally reduce physical demand while maintaining, or intentionally guiding, muscle activation toward a reference pattern or a clinically desired profile. Hereafter, we refer to the muscle activity observed during unassisted STS as the natural reference.

Previous studies on inducing muscle activity have typically relied on exoskeleton robots that directly regulate joint kinematics of a body segment closely related to the target muscles, for example, controlling elbow motion in upper-limb tasks (Teramae et al., 2025; Ueda et al., 2010). Extending such approaches to STS is nontrivial because STS is a whole-body, multi-joint transition in which posture and the distribution of mechanical demand across muscles change continuously over time (Doorenbosch et al., 1994). In principle, directly shaping muscle activity across the joints involved in STS would require coordinated multi-joint actuation and complex mechanical structures with multiple actuators, as implemented in lower-limb exoskeletons (Huo et al., 2022; Li et al., 2023). This motivates investigating whether higher-dimensional muscle activation patterns during STS can be guided using a mechanically simpler device that provides only a low-dimensional, indirect input.

However, the deployment of multi-actuator exoskeleton robots in daily-life settings can be challenging. Therefore, simpler and more practical devices are preferable for routine use (Rea and Ottaviano, 2018). Chair-type assistive devices offer an accessible alternative by supporting STS through seat motion during the initial rising phase, but due to their mechanical simplicity, such chairs typically provide partial and indirect assistance and may not fully regulate STS kinematics over the entire movement (O’Meara and Smith, 2005). Nevertheless, even limited seat motion up to seat-off can modify the global dynamics of the body (e.g., center-of-mass trajectory and joint velocities), which may in turn modulate multi-muscle activation patterns. This raises a key question: can a low-dimensional chair input, parameterized by a limited number of control variables relative to the large number of muscles involved in STS, be exploited to guide desired muscle activity profiles during STS?

**FIGURE 1:**
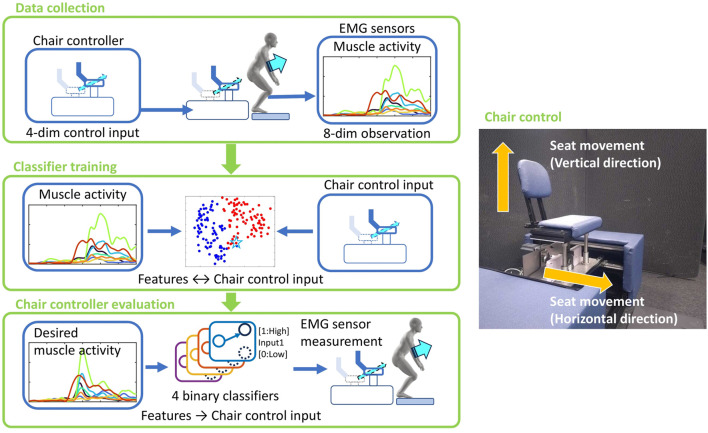
Proposed robotic chair assist strategy. The assistive chair, controlled by four variables, affects eight muscle activities toward various target profiles. We first verified its ability to modulate muscle activity by simultaneously recording control commands to the robotic device and the user’s muscle activity. To establish a control strategy, a classifier based on LASSO logistic regression was trained using the collected data to estimate control variables from muscle activity measured during natural STS. By employing this inverse model as a controller, desired muscle actiindependence ivity patterns were translated into seat-control commands.

To address this question, we collect paired data of chair control inputs and surface EMG during STS and learn an EMG-to-control parameter inference from EMG features to discrete chair control variables using $L_{1}$ -regularized logistic regression. We then evaluate, in an offline analysis, whether the estimated control variables select chair motions that yield muscle-activity features closer to a given target than alternative control combinations. An overview of the proposed system is presented in Figure 1.

The contributions of this study can be summarized as follows:

- We developed a method for inducing muscle activity using a simple two-DoF robotic chair during STS motion.
- We proposed a muscle activity feature extraction protocol and demonstrated that the extracted features are effective for estimating the movement commands of the robotic chair.
- We conducted muscle activity induction experiments with the robotic chair and found that muscle activities were successfully modulated to closely approximate the desired target profiles.

The rest of this article is organized as follows. Section 2 shows assistive devices and analyses for the STS motion, in addition to muscle inducement studies. Section 3 introduces our proposed methods and Sections 4–6 show the experimental configurations, results, and discussion, respectively. Finally, Section 7 concludes this study.

## 2 Related works

### 2.1 Assistive device for STS motion

Various assistive devices for STS motion have been proposed, including exoskeleton-type devices worn on the lower limbs (Li et al., 2023; Furukawa et al., 2022), devices that replicate the assistance of physical therapists (Lefévre et al., 2025), devices that lift the user’s entire body (Asai et al., 2024), and devices that correct whole-body movements (Matjačić et al., 2016). Chairs with assistive functions have also been explored as one category of such devices. For example, Narayan et al. developed a seat-elevation device with wheels (Narayan et al., 2020), while Wang et al. proposed a tilting seat mechanism to assist STS motion (Wang et al., 2021).

In this study, we employed an assistive chair we developed that allows the seat to move independently along the vertical and horizontal axes, with the velocity of each axis controlled separately (Kuroda et al., 2022). Previous studies have demonstrated that such chairs can reduce the burden of STS motion; however, they have not examined how assistive chairs influence muscle activity profiles or whether they can be used to achieve specific target patterns.

### 2.2 Analysis of STS motion

Various studies have investigated the biomechanics of STS motion (Dussault-Picard et al., 2024). Roebroeck et al. reported on the relationship between posture and muscle activity during STS (Roebroeck et al., 1994). Several studies have also examined ground reaction forces and accelerations in relation to muscle activity (Janssen et al., 2002).

Beyond direct analyses of individual muscles, other research has focused on grouping muscle activity into synergies (Tresch et al., 1999; d’Avella et al., 2003). Ranaldi et al. evaluated the number of synergies required for daily-life motions and characterized their features (Ranaldi et al., 2023). They identified the optimal number of synergies for different motion types as well as the corresponding muscle combinations. Furthermore, Ranaldi et al. analyzed the relationship between muscle synergies and the center of mass, proposing potential applications in which muscle synergies could be controlled based on center-of-mass dynamics (Ranaldi et al., 2025). With respect to muscle synergies in assisted STS motion, Wang et al. reported that the number of synergies increased when assistance was provided compared to unassisted STS (Wang et al., 2025). Their results showed that overall muscle activity trends were preserved with and without assistance, while reflecting variations related to the user’s posture and the applied support. These findings suggest that it may be possible to induce specific target muscle activities, provided that the desired patterns fall within the range of variability observed under assisted conditions.

Collectively, these studies highlight the complexity of STS motion and the importance of analyzing both individual muscle activity and higher-level synergies. However, little is known about how simple chair-type assistive devices can induce or reshape such muscle activity patterns.

### 2.3 Application of inducing muscle activity

Several previous studies have investigated methods for inducing muscle activity. These methods can be classified into two approaches: (i) evaluating the full waveform of muscle activity, and (ii) reducing muscle activity to lower-dimensional representations, such as absolute activation levels. As an example of the former, Teramae et al. proposed a rehabilitation-oriented system that induces muscle activity by controlling the user’s posture (Teramae et al., 2020). Torricelli et al. developed a system that guides delayed muscle activity by directly presenting the waveform of muscle activity to the user (Torricelli et al., 2020). The latter approach focuses on low-dimensional representations. For instance, Teramae et al. proposed a posture-control system for inducing muscle activity in rehabilitation contexts (Teramae et al., 2025), while Ueda et al. set multiple target muscle activities and evaluated how closely induced patterns matched these targets using an exoskeleton robot applied to the upper arm (Ueda et al., 2010).

These prior studies primarily targeted continuous movements or tasks in which the user’s posture remained relatively constant. By contrast, inducing short-term muscle activity patterns during posture transitions using a simple two-DoF assistive chair represents a novel contribution of this study.

## 3 Methods

**FIGURE 2:**
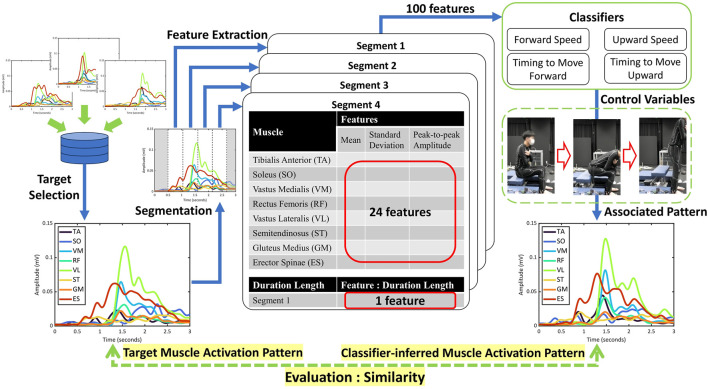
Workflow of the proposed method. EMG-based muscle activation during STS is segmented into four phases using the torso tilt angle and the seat-off event (see Section 4.3). From each phase, one temporal feature (segment duration) and three EMG features per muscle (mean, standard deviation, and peak-to-peak amplitude) are extracted and concatenated (100 features per trial). Four binary classifiers then estimate the chair control variables (forward/upward speed and onset timing) from the feature vector.

Figure 2 illustrates the overall workflow of this study. First, muscle activity data were collected and processed to extract features. Next, Lasso logistic regression was used to estimate the control variables of the assistive chair from these features (see Section 3.1). Finally, the estimated control variables were evaluated for their potential to induce the target muscle activity in an offline manner (see Section 3.2).

### 3.1 Control variable estimation for assistive chair

Our assistive chair controls seat motion through four control variables: the vertical and horizontal movement velocities, and the onset timing of each movement. Two levels–fast and slow–were defined for both the speed and timing along each axis, yielding 16 combinations of control variables (two levels for each of four variables). These variables are estimated from features extracted from the measured muscle activities.

To estimate the binary states (fast or slow) of four control variables, we employed Lasso logistic regression. We selected Lasso because its $L_{1}$ -regularization term encourages sparsity in the model coefficients, effectively performing feature selection among the high-dimensional muscle activity features $x_{i}\in R^{p}$ . Let ${\left\{\left(x_{i},y_{i}\right)\right\}}_{i=1}^{N}$ denote the dataset of input features and corresponding chair control variables. We model the relationship using logistic regression, where $y_{i}=1$ indicates that the i -th control variable is in the “fast” state, and $y_{i}=0$ indicates the “slow” state. The probability that the i -th control variable takes the “fast” state given the input features $x_{i}$ is

$${\hat{y}}_{i}=\frac{1}{1+\exp\left(-\left(\beta_{0}+x_{i}^{T}\beta_{1}\right)\right)}.$$ (1)

The model parameters $\beta_{0}$ and $\beta_{1}$ are estimated by minimizing the regularized logistic loss Tibshirani (1996); Vidaurre et al. (2013):

$$\hat{\beta_{0}},{\hat{\beta }}_{1}=\arg\min_{\beta_{0},\beta_{1}}\left\{-\overset{N}{\underset{i=1}{\sum }}\left[y_{i}\log{\hat{y}}_{i}+\left(1-y_{i}\right)\log\left(1-{\hat{y}}_{i}\right)\right]+\lambda \overset{p}{\underset{j=1}{\sum }}|\beta_{j}|\right\},$$ (2)

where λ≥0 is a regularization parameter. To implement the classification, we used a ‘lassoglm’ function provided by MATLAB^1^. The control variable y is estimated from the features x derived from muscle activities as described in Section 4.3. In this study, each input feature vector $x_{i}$ had p=100 dimensions, corresponding to the extracted muscle activity feature. In addition, we used one classifier for each control variable, resulting in a total of four classifiers.

### 3.2 Evaluation of classifier-inferred muscle activity

In this study, we conducted an offline evaluation to verify whether the estimated control variables can induce the target muscle activity features. First, the dataset consisting of muscle activity features and the corresponding control variables was divided into training and test sets. Using the training data, we constructed a classifier to estimate control variables from muscle activity features, as described in Subsection 3.1. Second, the muscle activity features in the test data were regarded as the target features and input into the trained classifier to estimate the corresponding control variables. Third, by averaging the muscle activity feature samples associated with each estimated control variable in the training data, we obtained a representative feature vector for each variable. This vector was defined as the classifier-inferred muscle activity. Finally, we evaluated the potential of the estimated control variables to induce the target muscle activity by computing the similarity between the target and classifier-inferred feature vectors.

The similarity between the target and classifier-inferred features $S_{\text{INF}}$ is evaluated in the feature space as the Euclidean distance between the 100-dimensional feature vectors f , normalized by the sum of the distances across all possible control variable combinations:

$$S_{\text{INF}}=\frac{\sqrt{\sum_{i=1}^{100}{\left(f_{inferred_{i}}^{\mathrm{est}}-f_{target_{i}}\right)}^{2}}}{\sum_{n=1}^{16}\sqrt{\sum_{i=1}^{100}{\left(f_{inferred_{i}}^{n}-f_{target_{i}}\right)}^{2}}}$$ (3)

For comparison, we also computed an empirical baseline score, $S_{\text{NT}}$ , where the subscript NT denotes “Non-Target”. This score was obtained by evaluating the distances between the target muscle activity features and the classifier-inferred muscle activity features corresponding to all 15 non-target control variable combinations. This baseline reflects the similarity that would be obtained if the estimated control variable were incorrectly assigned to any of the other combinations. By comparing $S_{\text{INF}}$ with this empirically derived baseline, we assessed whether the estimated control variables produced muscle activity patterns that were more similar to the target features than those associated with the remaining control combinations.

### 3.3 Feature separability

In addition to classification performance, we evaluated the separability of the feature space for each control variable. Feature separability, denoted as η , was used as a quantitative index for the binary classification (Otsu, 1979). η is defined as a ratio of the within-class variation $\sigma_{W}^{2}$ and the between-class variation $\sigma_{B}^{2}$ , thus η is calculated as follows:

$$\eta =\frac{\sigma_{B}^{2}}{\sigma_{W}^{2}}$$ (4)

Here, the higher η means the lower difficulty of the classification. In this study, we have the same amounts of data labeled as 0 (the slow speed or timing) and 1 (the fast speed or timing), therefore $\sigma_{W}^{2}$ and $\sigma_{B}^{2}$ are calculated as follows:

$$\sigma_{W}^{2}=\frac{\sigma_{0}^{2}+\sigma_{1}^{2}}{2}$$ (5)

$$\sigma_{B}^{2}=\frac{{\left(\mu_{0}-\mu_{\mathrm{total}}\right)}^{2}+{\left(\mu_{1}-\mu_{\mathrm{total}}\right)}^{2}}{2}$$ (6)

where $\sigma_{0}$ , $\sigma_{1}$ , $\mu_{0}$ , $\mu_{1}$ , and $\mu_{\mathrm{total}}$ represent the variance and the average of data labeled as 0 and 1, and the overall average. This study evaluated not all input features but the features that each classifier utilized. Here, the non-zero elements of the β in Equation 2 of each classifier were the evaluation targets.

## 4 Experimental setup

### 4.1 Control variables

We obtained 16 combinations of the four control variables by assigning two levels to each. For the starting timing of seat movement, two cases are defined: (1) a fixed waiting time of 0.3 s after the participant begins tilting their torso, and (2) no waiting (0 s). These values were determined based on the estimated time required to perform the STS motion in a preliminary experiment.

For the seat movement speed, we selected two values: 0.08 m/s and 0.22 m/s, which are close to the lower and upper limits of the mechanically stable operating range of the assistive chair. Our previous study demonstrated that the device could control its speed from 0.05 m/s to 0.25 m/s, allowing it to adapt to various assistance speeds for sit-to-stand assists (Kuroda et al., 2022).

The torso tilt angle was measured using an IMU (Microstrain 3DM-GX3-25) attached to the participant’s chest. The duration of the STS motion was defined as the period during which the torso was tilted forward by more than 10° from the initial seated posture. The seat-off timing was defined as the time at which the reaction force from the seat dropped below 5% of the initial pressure, which approximately corresponds to the participant’s body weight. The upward and forward movements of the chair were set to 15 cm, based on the mechanical constraints of the assistive chair and previous studies.

### 4.2 Participant

Six healthy adult males (age: 29.3 ± 5.0 years; height: 175.5 ± 5.5 cm; weight: 75.0 ± 12.2 kg) participated in the study. The experimental protocol was approved by the human research ethics committee of RIKEN (RIKEN-W1-2024–018), and written informed consent was obtained from all participants.

### 4.3 Data collection

**FIGURE 3:**
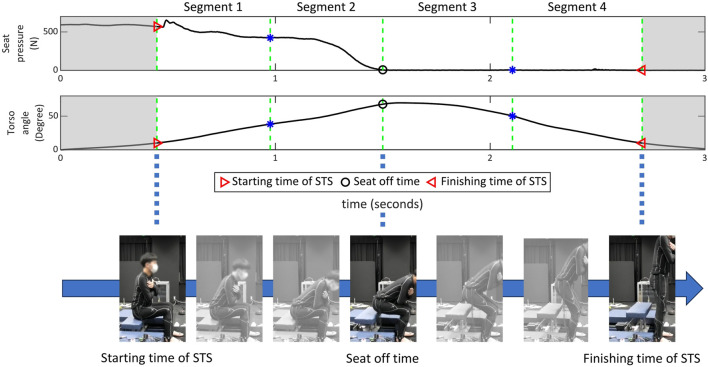
Example sit-to-stand (STS) trial performed with the assistive chair and the temporal segmentation used in this study. The top panels show the seat reaction force and torso tilt angle over time. The bottom panel illustrates the four-phase STS segmentation: red triangles indicate STS onset and completion, the black circle indicates seat-off, and the remaining markers indicate intermediate phase boundaries.

For each of the 16 control variable sets, 10 sit-to-stand (STS) motion trials were recorded per participant. The order of the control variable sets was randomized for each participant, and several minutes of rest were provided between sets to minimize fatigue. During each trial, we measured muscle activity using EMG, torso angle with an IMU, and reaction force from the seat. The collected motion data were segmented based on the torso tilt angle and seat-off timing, as illustrated in Figure 3 (Boukadida et al., 2015). For classifier training, 70% of the trials were used as the training set, and the remaining 30% were used for evaluation.

**FIGURE 4:**
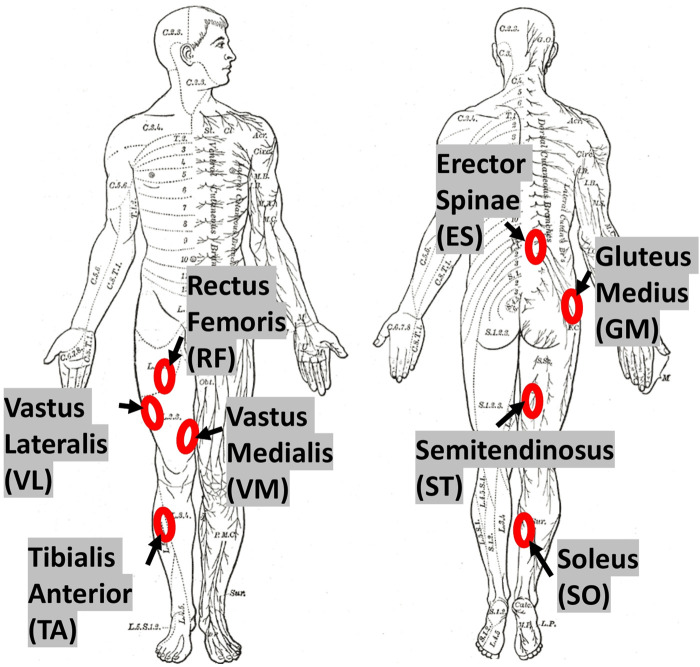
Locations of the EMG measurement sites (target muscles) on the participant’s right side: Tibialis Anterior (TA), Soleus (SO), Vastus Medialis (VM), Rectus Femoris (RF), Vastus Lateralis (VL), Semitendinosus (ST), Gluteus Medius (GM), and Erector Spinae (ES). Anatomical drawings adapted from Gray’s Anatomy (Gray and Lewis, 1918).

**FIGURE 5:**
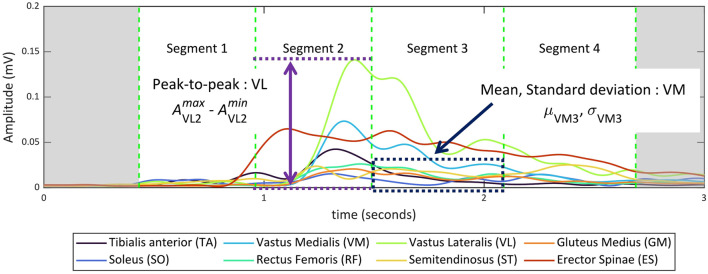
Three features from muscles. The mean, standard deviation, and peak-to-peak amplitude of the muscle activation $\left(\mu_{iN}\right)$ , standard deviation $\left(\sigma_{iN}\right)$ , and peak-to-peak amplitude $\left(A_{iN}^{\max}-A_{iN}^{\min}\right)$ were obtained from muscle i and segment N .

We measured eight muscle activities on the right side of the body: Tibialis Anterior (TA), Soleus (SO), Vastus Medialis (VM), Rectus Femoris (RF), Vastus Lateralis (VL), Semitendinosus (ST), Gluteus Medius (GM), and Erector Spinae (ES), as shown in Figure 4. These muscle activities were recorded using surface electromyography with handmade bipolar electrodes. The raw EMG signals were processed as follows: First, a bandpass filter (40–400 Hz) was applied; then the signal was rectified and low-pass filtered at 4 Hz; finally, the processed signals were converted into the muscle activation data (Yang et al., 2019). All filters were implemented as fourth-order Butterworth filters. For each STS segment, we extracted one temporal feature (segment duration) to capture the timing and speed of the motion. In addition, three features per muscle were obtained: the mean $\left(\mu_{iN}\right)$ , standard deviation $\left(\sigma_{iN}\right)$ , and peak-to-peak amplitude $\left(A_{iN}^{\max}-A_{iN}^{\min}\right)$ from activation A of muscle i at segment N , as shown in Figure 5. The mean reflects the overall muscle activation level, the standard deviation captures variability in the activation during the motion, and the peak-to-peak amplitude indicates the range of muscle contraction. These features were selected to provide a concise representation of both the intensity and temporal characteristics of muscle activity during the STS motion. Thus, we had 25 features per STS segment. Since each STS motion consisted of four segments, the input feature vector $x_{i}$ had 100 elements per motion (see also Table 1). All extracted features were standardized before being used as input for the classifiers as shown in Equation 1.

**TABLE 1:** Feature composition per STS repetition.

Category	Details	Count
Muscles	# of channels	8
Time-segmented EMG signals	# of movement segments	4
Features per muscle per segment	Peak-to-peak, mean, variance	3
EMG features	3×8×4	96
Segment duration features	1 per segment	4
Total	96 + 4	100

## 5 Results

### 5.1 Performance of control variable estimation

We evaluated the performance of the Lasso logistic regression classifiers in estimating the binary states (fast or slow) of the four control variables: upward and forward velocities, and their onset timings. Each classifier corresponds to one control dimension of the assistive chair, and their comparison clarifies which aspects of chair control can be reliably predicted from muscle activity.

Table 2 shows the classification performance across all participants, expressed as the mean and standard deviation of precision, recall, and F-score. Precision and recall are presented to complement the F-score and provide a more detailed picture of classifier tendencies. Overall, classifiers for speed estimation achieved higher performance than those for timing estimation. Among them, the classifier for upward speed showed the highest F-score, while that for upward timing exhibited the lowest. These results suggest that muscle activity features associated with vertical velocity are relatively consistent and discriminative across participants, whereas timing-related features are more variable and sensitive to individual differences. This comparison indicates that velocity-related control variables can be estimated more robustly than timing-related ones, providing valuable insight for the design of adaptive assistance strategies. Although there are differences among control variables, all classifiers achieved reasonably high F-scores, indicating that the Lasso logistic regression can reliably predict the chair control states from muscle activity features.

**TABLE 2:** Classification performance (mean ± SD across participants) for each control variable.

Control variable	Precision	Recall	F-score
Forward speed	0.96 ± 0.04	0.96 ± 0.04	0.96 ± 0.04
Upward speed	1.00 ± 0.01	0.99 ± 0.01	0.99 ± 0.00
Timing to move forward	0.88 ± 0.06	0.91 ± 0.05	0.89 ± 0.04
Timing to move upward	0.83 ± 0.10	0.81 ± 0.12	0.82 ± 0.11

### 5.2 Feature selection on estimation

**FIGURE 6:**
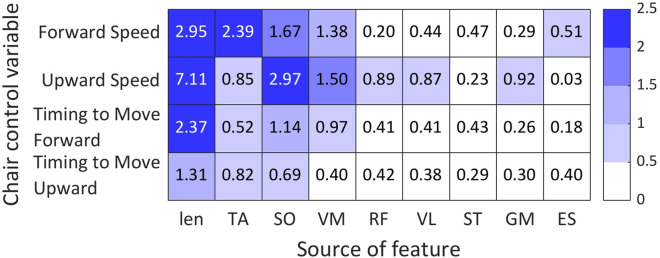
Mean absolute LASSO coefficient magnitudes $\left(\left|\beta_{1}\right|\right)$ for the four logistic-regression classifiers, averaged across all participants. Rows correspond to the chair-control variables (Forward Speed, Upward Speed, Timing to Move Forward, and Timing to Move Upward), and columns indicate the feature source. “len” denotes segment-duration features, and “TA”, “SO”, “VM”, “RF”, “VL”, “ST”, “GM”, and “ES” denote EMG-derived features from the corresponding muscles. Each cell reports the mean of the absolute coefficients associated with that feature source; larger values indicate greater contribution to the corresponding control-variable estimation.

We further examined which muscle activity features contributed to the estimation weights. Figure 6 shows a heatmap of averaged Lasso weights across all participants, with the horizontal axis representing features from all muscles and the vertical axis representing the four control variables. For the speed-related parameters, distinct patterns were observed. For Forward Speed, large weights were assigned to the TA, SO, and VM, while other muscles contributed only minimally. For Upward Speed, SO, VM, TA, and RF showed the highest weights, whereas VL, GM, and ES exhibited small contributions. These findings indicate that both forward and vertical movement estimation depend primarily on ankle and knee extensor–flexor muscle activity. It is assumed that the chair velocity generates inertial assistance, thereby reducing the mechanical workload required to elevate the body, work that would otherwise be primarily produced by the agonist muscles responsible for ankle and knee extension (TA, VM, RF, and VL). In addition, this externally induced inertia appears to elicit activation of the antagonist muscle for ankle extension (SO), likely contributing to postural stabilization against the imposed inertial forces.

In contrast, the two timing-related control variables (Timing to Move Forward and Timing to Move Upward) showed a more uniform weight distribution across muscles. Although TA, SO, and VM tended to exhibit slightly larger weights than other muscles, no single muscle dominated the contribution. This indicates that timing estimation relies on a more balanced integration of information from multiple muscle groups, rather than being driven by a particular agonist muscle. The features were mainly derived from the muscle’s amplitude, and timing information could be omitted during extraction. Therefore, the classifications of timing parameters had lower performances than those of speed parameters.

Overall, the observed weight patterns are consistent with known biomechanical characteristics of the STS movement, and they also align with the estimator’s classification performance, indicating that the model relies on physiologically meaningful features.

### 5.3 Feature separability

**FIGURE 7:**
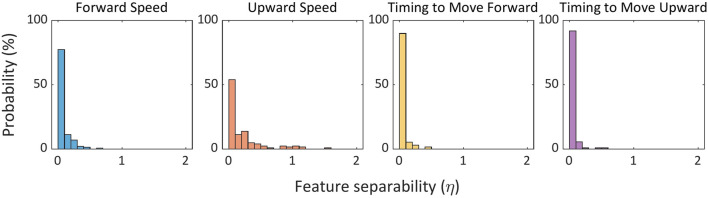
Histograms of feature separability (η) for each chair control variable, aggregated across all participants. For each classifier, η was computed for the subset of features used by the model (i.e., features with non-zero coefficients), and the histogram shows the resulting probability distribution of η . Larger η indicates better separation between the two classes, whereas η<1 indicates that within-class variability dominates over between-class differences, suggesting a more challenging classification problem.

Figure 7 shows the distribution of separability scores across participants and control variables. The results indicate that, although the classifiers achieved reasonably high F-scores, the separation between classes in the feature space was moderate, particularly for timing-related variables. This suggests that classification was nontrivial and relied on subtle differences in EMG patterns rather than on clearly separable clusters as shown in Equations 5, 6. The moderate separability also provides a potential explanation for the relatively lower performance of timing-related classifiers compared to those for velocity estimation.

### 5.4 Classifier-inferred muscle activity

**FIGURE 8:**
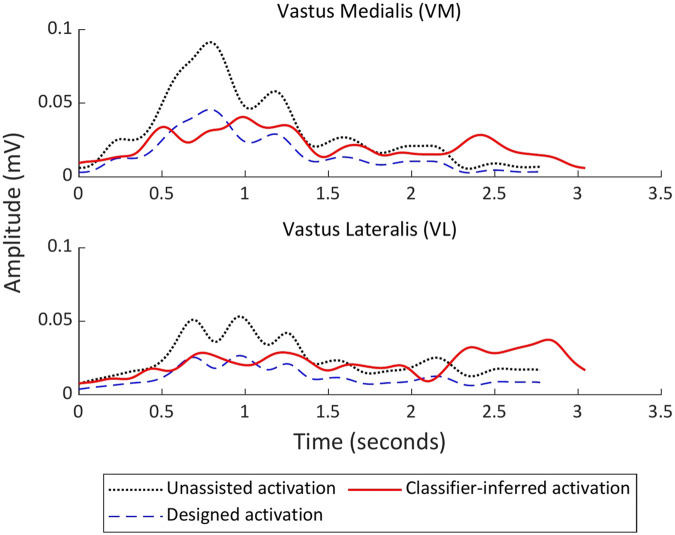
Comparison of muscle activation patterns during a sit-to-stand (STS) trial (representative example from one participant), focusing on the Vastus Medialis (VM) and Vastus Lateralis (VL). The designed target pattern aims to reduce VM and VL activation relative to the unassisted STS baseline. Black dotted lines denote unassisted activation, blue dashed lines denote the designed (target) activation, and red solid lines denote the classifier-inferred activation measured when applying the chair-control variables estimated for the target.

We analyzed how the assistive char influences muscle activity during the STS motion in an offline manner. Figure 8 illustrates a representative example obtained from one participant, focusing on the Vastus Medialis (VM) and Vastus Lateralis (VL), which are agonist muscles for knee extension and are expected to be directly affected by chair assistance. The designed pattern represents the intended reductions in VM and VL activity, while the classifier-inferred muscle activity pattern shows the actual muscle activation achieved when using the assistive chair. For reference, the unassisted pattern shows the baseline activation during an STS motion without assistance. The root mean square (RMS) values of filtered EMG signals across all eight measured muscles were 0.14 mV, compared to 0.17 mV in the unassisted motion, indicating that the assistive chair reduced target muscle activity without evoking significant compensatory activation in other muscles.

**FIGURE 9:**
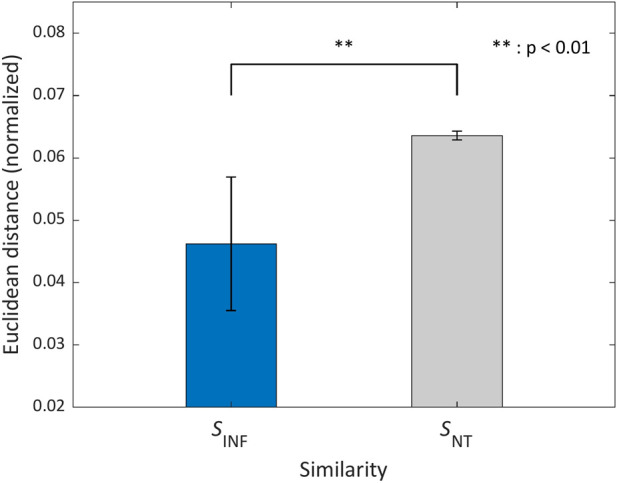
Normalized Euclidean distance in feature space between the target and classifier-inferred muscle activity feature vectors, aggregated across all participants. The blue bar shows $S_{\text{INF}}$ , i.e., the distance obtained using the control-variable combination estimated for the target features. The gray bar shows the empirical non-target baseline $S_{\text{NT}}$ , computed from the remaining 15 (non-target) control-variable combinations. Error bars indicate the standard deviation across participants. All distances were normalized by the sum of distances over all 16 candidate control-variable combinations (see Section 3.2). ** p<0.01 .

In addition to the representative activation patterns, we evaluated how accurately the estimated control variables reproduced the target muscle activity features using the similarity metric $S_{\text{INF}}$ . Figure 8 compares unassisted muscle activation, the designed target activation, and the classifier-inferred (offline feature-space) activation. This figure is intended to illustrate qualitative trends in the data. Quantitative conclusions regarding the similarity between muscle activation patterns are based on the feature-space similarity metrics ( $S_{\text{INF}}$ and $S_{\text{NT}}$ ), rather than this figure alone. Figure 9 compares $S_{\text{INF}}$ with the empirical baseline $S_{\text{NT}}$ , which was computed using all 15 non-target control variable combinations. Across participants, $S_{\text{INF}}$ was significantly lower than $S_{\text{NT}}$ , indicating that the estimated control variables classifier-inferred muscle activity patterns substantially closer to the target features than those associated with incorrect control combinations (mean ± SD: $S_{\text{INF}}$ = 0.046 ± 0.011, $S_{\text{NT}}$ = 0.064 ± 0.001). Statistical analysis using a Wilcoxon rank-sum test confirmed a significant difference between the two conditions ( |z| = 17.78, p < 0.01). These results demonstrate that the proposed estimation framework reliably identifies control variables capable of inducing the desired muscle activity features.

## 6 Discussion

In this study, we investigated how an assistive chair affects muscle activity during the sit-to-stand motion. We also examined whether muscle activation patterns can be reliably predicted from EMG features. Our results show that velocity-related control variables of the chair can be estimated reliably from muscle activity, whereas timing-related variables exhibit greater variability across participants. This indicates that certain aspects of chair control are more consistently reflected in EMG signals, providing a stable basis for adaptive assistance strategies.

The analysis of the feature contributions revealed that, for upward speed, the VM and VL were dominant contributions, whereas other control variables did not show clear feature dominance. These findings align with the higher classification performance observed for upward speed, suggesting that distinct, classifiable EMG patterns facilitate reliable prediction. In contrast, the moderate separability of features calculated by Equation 4 for timing-related variables highlights the difficulty in decoding these parameters, which likely explains the relatively lower performance of the corresponding classifiers. Importantly, the proposed system successfully inferred muscle activity patterns that closely matched the target profiles, particularly VM and VL, without causing unintended activation in other muscles. This demonstrates that the simple classifier-based estimation method effectively captured the mapping between EMG activity and control variables. However, for control variables with lower classification performance, such as timing, the classifier-inferred muscle activity deviated more from the target, underscoring the dependency of muscle activity induction on accurate control variable estimation. These evaluations were performed offline, and real-time feedback, as well as individual differences, may affect muscle activity induction in practice, warranting further study.

The similarity metric $S_{\text{INF}}$ , defined as a normalized Euclidean distance in the 100-dimensional EMG feature space as shown in Equation 3, provides a statistical measure of similarity between multi-muscle activation patterns under different control parameters. It does not directly correspond to a physiological or biomechanical quantity such as muscle force or joint torque. Because the feature vector combines heterogeneous quantities (peak-to-peak amplitude, mean, variance, and segment duration), all features were normalized to zero mean and unit variance prior to distance computation. Therefore, $S_{\text{INF}}$ reflects relative deviations across muscles and movement phases on a comparable scale, rather than being dominated by any single feature type. The observed reductions in $S_{\text{INF}}$ relative to $S_{\text{NT}}$ indicate that the estimated control parameters yield EMG feature patterns that are closer to the target coordination patterns across muscles and phases than non-target control combinations. However, EMG signals are inherently non-stationary and heterogeneous, and the physiological or biomechanical interpretation of $S_{\text{INF}}$ remains limited. Establishing a direct mapping from these distance values to neuromuscular or biomechanical function is a topic for future research.

Several limitations of this study should be considered. Only a single functional task (sit-to-stand) was investigated with a limited number of homogeneous participants, in terms of health and gender condition. The investigation was conducted offline and relied on averaged training data for prototype construction. While strict data leakage did not occur, more rigorous validation schemes–such as leave-one-subject-out cross-validation and real-time experimental validation–would be necessary to further reduce potential optimistic bias. Generalization to a broader population and other types of movement also remains to be validated. Additionally, the design targets and evaluation criteria may need adaptation for different assistive applications. At present, the control input is restricted to binary values, incorporating more fine-grained (multi-level) control inputs is left for future investigation.

Overall, our findings suggest that muscle-driven prediction and control of assistive devices is feasible and can achieve targeted muscle modulation. These results provide a foundation for developing more effective, individualized assistive robots that leverage neuromuscular signals for adaptive support.

## 7 Conclusion

This study proposed a data-driven approach to induce target muscle activity using a classifier-based inference of assistive control parameters. A set of control variables was exhaustively tested to construct a training dataset linking control inputs to corresponding muscle activity patterns. Classifiers trained via Lasso logistic regression were able to predict control variables from target muscle activity, with varying performance depending on the variable type. In particular, classifiers for speed-related variables exhibited higher accuracy and clearer feature selection patterns, which contributed to the successful induction of muscle activity.

Offline evaluation showed that the proposed system inferred muscle activity patterns more closely matching the target profiles than random parameter selection. Importantly, the system was able to selectively reduce load on the intended muscles (e.g., VM and VL) without eliciting unintended activation in other muscles. Feature space analysis further suggested that subtle EMG patterns underlie the decoding of control variables, particularly for timing-related parameters, highlighting challenges and opportunities for future improvements.

These findings suggest the potential of inverse muscle activity mapping for various future real-time assistive control systems, ranging from STS assistance to various activities of daily living. However, several limitations should be considered, including variable interdependence, individual variability, reliance on a single functional task, and the offline nature of evaluation. Future studies should aim to induce a broader range of muscle activity patterns while further improving similarity to the target profiles, including participants with various characteristics, e.g., gender, age, and ability. Evaluating muscle activity in higher-dimensional or more direct representations, rather than relying solely on low-dimensional features, may further enhance the performance and generalizability of the system. Finally, the real-time system should be implemented with robust feature extraction that covers these findings and limitations.

## Data Availability

The raw data supporting the conclusions of this article will be made available by the authors, without undue reservation.

## Footnotes

1
https://www.mathworks.com/help/stats/lassoglm.html, Accessed on 4 Jul 2025.
